# Usefulness of the G8 Geriatric Assessment Tool as a Prognostic Factor in Gemcitabine Plus Nab-paclitaxel Combination Therapy for Elderly Patients with Pancreatic Cancer

**DOI:** 10.31662/jmaj.2022-0086

**Published:** 2022-09-20

**Authors:** Makoto Kadokura, Yuki Mori, Yumi Takenaka, Hiroki Yoda, Tomoki Yasumura, Keisuke Tanaka, Fumitake Amemiya

**Affiliations:** 1 Department of Gastroenterology, Kofu Municipal Hospital, Kofu, Japan

**Keywords:** Pancreatic cancer, gemcitabine plus nab-paclitaxel, prognostic factor, G8 geriatric assessment tool, neutrophil-lymphocyte ratio

## Abstract

**Introduction:**

The usefulness of various prognostic factors for advanced pancreatic cancer (APC) has been reported, but the number of elderly patients in these studies is disproportionately fewer than those in general practice. This study aimed to examine the prognostic factors for elderly patients with APC receiving gemcitabine plus nab-paclitaxel (GnP) considering the G8 geriatric assessment tool.

**Methods:**

We retrospectively analyzed 77 elderly (≥65 years old) patients with APC who received GnP as first-line chemotherapy at our hospital. We used the receiver operating characteristic curve to set the optimal cutoff value for G8. Univariate and multivariate Cox regression models were applied to study independent prognostic factors.

**Results:**

The progression-free survival was 5.5 months, and the overall survival (OS) was 12.0 months in all patients. The most optimal cutoff of G8 was 10.5. OS of G8 ≥10.5 patients was superior to that of G8 <10.5 patients (18.5 versus 8.0 months). Multivariate analysis showed that Eastern Cooperative Oncology Group performance status 1 (hazard ratio [HR] 3.00, p = 0.02), neutrophil-lymphocyte ratio ≥3.9 (HR 2.73, p = 0.03), and G8 geriatric assessment <10.5 (HR 5.38, p < 0.001) were independent negative prognostic factors.

**Conclusions:**

G8 is useful for predicting prognoses in elderly patients with APC receiving GnP.

## Introduction

Pancreatic cancer is still the most lethal malignancy among common tumors and the seventh leading cause of cancer-related death worldwide^(1)^. Approximately 45,750 patients died from this disease in 2019 in the United States ^(2)^. In Japan, over 40,000 patients are diagnosed with pancreatic cancer annually, with most patients dying, leading to estimated 35,000 deaths ^(3)^. The mortality rates closely follow incidence rates because of poor prognoses.

Recently, gemcitabine plus nab-paclitaxel (GnP) treatment has been recommended as standard therapy for patients with advanced pancreatic cancer (APC). This regimen showed superiority in survival versus gemcitabine alone in the large phase III MPACT trial ^(4)^. In the MPACT trial, subgroup analysis revealed a significant survival advantage of GnP versus gemcitabine alone in patients <65 years old. However, this advantage was not found in patients ≥65 years old. Moreover, the long-term follow-up data of the MPACT trial determined age as a significant independent predictor of survival ^(5)^. Pancreatic cancer incidence increases with age ^(6)^. The age-standardized incidence rate in patients >75 years old tends to be higher in Japan than in other countries ^(7)^. Elderly cancer patients represent a heterogeneous group with different biological, functional, and psychosocial characteristics; hence, revealing a prognostic indicator of efficacy for this elderly age group is important.

In elderly patients with APC, several prognostic factors, including the neutrophil-lymphocyte ratio (NLR) ^(8), (9), (10), (11), (12), (13)^, modified Glasgow prognostic score (mGPS) ^(8), (13), (14)^, and Geriatric Nutrition Risk Index (GNRI) ^(15)^, have been proposed. The International Society of Geriatric Oncology recommended evaluating elderly cancer patients with a geriatric assessment (GA) to detect problems not readily identified by routine physical examinations or medical history to predict cancer treatment-related toxicities and survival and assist in cancer treatment decisions ^(16)^. The G8 screening tool comprises seven items on food intake, weight loss, mobility, neuropsychological problem, body mass index, prescription drugs, and self-perception of health (Table 1) ^(17)^. A total score ranges from 0 (poor score) to 17 (good score), and a total score of ≤14 is considered abnormal. However, APC is frequently associated with anorexia, low nutrition, and weight loss. Therefore, it remains unclear whether G8 classification into two groups with a cutoff value of 14 is adequate. Gebbia et al. ^(18)^ reported that the median survival of G8 fit (>14) patients was superior to that of G8 vulnerable patients (6.5 versus 4 months), although the difference was not statistically significant. Thus, this study aimed to clarify whether G8 has an independent prognostic value for elderly patients with APC receiving GnP and what is the optimal cutoff value for predicting prognosis in elderly patients with APC.

**Table 1.** G8 Geriatric Assessment Tool.

Item	Score
1. Has food intake declined over the past 3 months due to loss of appetite, digestive problems, chewing, or swallowing difficulties?	0 = severe decrease in food intake
	1 = moderate decrease in food intake
	2 = no decrease in food intake
2. Weight loss during the last 3 months?	0 = weight loss >3 kg
	1 = does not know
	2 = weight loss between 1 and 3 kg
	3 = no weight loss
3. Mobility?	0 = bed or chair bound
	1 = able to get out of bed/chair
	but does not go out
	2 = goes out
4. Neuropsychological problems?	0 = severe dementia or depression
	1 = mild dementia
	2 = no psychological problems
5. Body mass index?	0 = BMI <19
	1 = BMI 19 to <21
	2 = BMI 21 to <23
	3 = BMI ≥23
6. Takes more than three prescription drugs per day?	0 = yes
	1 = no
7. In comparison with other people of the same age, how does the patient consider his/her health status?	0.0 = not as good
	0.5 = does not know
	1.0 = as good
	2.0 = better
8. Age	0: >85
	1: 80-85
	2: <80
Total score	0-17

## Materials and Methods

### Study population and data collection

We retrospectively investigated the data of elderly (≥65 years old) patients with APC who received GnP chemotherapy between January 2015 and June 2021 at Kofu Municipal Hospital. We retrieved patient records from a maintained database at our hospital and performed a systematic retrospective review of patient diagnosis, treatment, and laboratory data. Laboratory assessment at baseline included complete blood cell count, serum biochemistry, and serum tumor markers levels, such as carcinoembryonic antigen (CEA) and carbohydrate antigen 19-9 (CA19-9). The institutional review board of Kofu Municipal Hospital approved this study (approval code R2-3). Participants provided informed consent in the form of opt-out on the website.

### Prognostic variables

We examined the presence or absence of distant metastasis, Eastern Cooperative Oncology Group (ECOG) PS, CEA, CA19-9, mGPS, NLR, and GNRI as potential prognostic factors concerning previous reports. mGPS score was calculated as follows: 2, elevated c-reactive protein (CRP) (≥10 mg/L) and low albumin (<3.5 g/dL); 1, elevated CRP only; and 0, normal CRP (<10 mg/L). The NLR was calculated as the neutrophil percentage value divided by the lymphocyte percentage value. Based on our previous study ^(13)^, high NLR was defined as 3.9. In the same way, based on a previous study dealing with elderly patients treated with GnP ^(15)^, a cutoff value of GNRI was defined as 86. Provided present infection and/or jaundice, these parameters were measured after the symptoms had been relieved.

### G8 assessment

In our hospital, all gastrointestinal cancer patients have been routinely evaluated for G8 by physicians since January 2015 (Table 1). Based on the results of MPACT ^(4)^ and other clinical trials ^(15), (19)^ conducted in Japan, we used Receiver Operating Characteristic curve with the survival of more than 1 year as the outcome to set the optimal cutoff value for predicting prognosis.

### Treatment and related evaluation

GnP chemotherapy, including schedules and standard doses, was adjusted at the attending physician’s discretion based on adverse events or each patient’s general condition. Tumor response was assessed according to the Response Evaluation Criteria in Solid Tumors criteria by computed tomography scans at intervals of at least 3 months. Evaluation procedures were performed ahead of schedule if the patient’s general condition worsened or severe adverse events occurred. Toxicity was graded according to the Common Terminology Criteria for Adverse Events.

### Statistical analysis

Survival curves were estimated according to the Kaplan-Meier method, and differences were evaluated with a log-rank test. Variables that achieved statistical significance (p < 0.05) in univariate analysis were used for multivariate Cox regression analysis to determine significant independent factors. We also calculated the hazard ratio (HR) and 95% confidence intervals (CI). Significance was considered as a *p* value < 0.05. All statistical analyses were performed using EZR (Saitama Medical Center, Jichi Medical University, Saitama, Japan), a graphical user interface for R (the R Foundation for Statistical Computing, Vienna, Austria).

## Results

### Patient characteristics

A total of 77 elderly patients with APC were examined. Table 2 presents patient demographics. The median age was 72 years. A total of 56 patients had distant metastasis, and the remaining 21 patients had APC locally. Twenty-three (29.9%) patients started chemotherapy with a reduced dose of gemcitabine and nab-paclitaxel.

**Table 2.** Patient Characteristics.

Category		N (%) (n = 77)
Gender (%)		
	Male	47 (61)
	Female	30 (39)
Age at chemotherapy (years)	
	Median (range)	72 (65-91)
ECOG performance status	
	0	67 (87)
	1	10 (13)
Body mass index	
	Median (range)	21.2 (17.2-30.5)
Disease status	
	Locally advanced	21 (27.3)
	distant metastasis	56 (72.7)
Metastatic site	
	Liver	35 (45.5)
	Peritoneum	15 (19.5)
	Number of metastatic sites 2≥	22 (28.6)
G8 geriatric screening	
	Median (range)	10.5 (6.5-15.5)
	≤10	34 (44.2)
	≥10.5	43 (55.8)
Geriatric nutrition risk index	
	Median (range)	97.4 (75.8-114.5)
	≤86	66 (85.7)
	>86	11 (14.3)
Neutrophil-lymphocyte ratio	
	Median (range)	2.66 (0.72-13.6)
	<3.9	65 (84.4)
	≥3.9	12 (15.6)
Modified Glasgow Prognostic Score		
	0	59 (76.6)
	1-2	18 (23.4)
Carcinoembryonic antigen (ng/ml)		
	<5.0	36 (46.8)
	≥5.0	41 (53.2)
Carbohydrate antigen 19-9 (U/ml)		
	<37.0	20 (26)
	≥37.0	57 (74)

### Outcome

**Figure 1.**
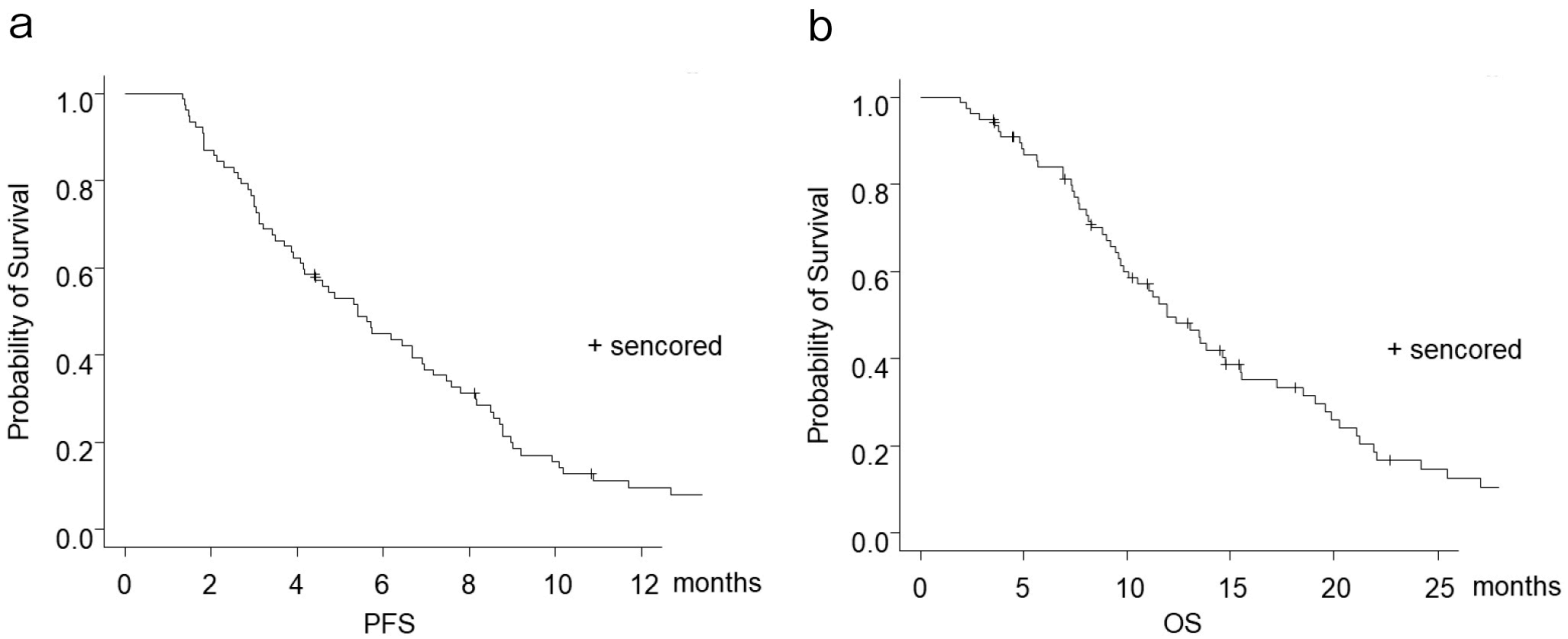
Kaplan-Meier estimates for progression-free survival (a); for overall survival (b).

None achieved complete response. Partial response was identified in 17 patients (22.1%), and stable disease was reported in 29 patients; hence, the disease control rate was 59.7%. The progression-free survival was 5.5 months (Figure 1a), and the overall survival (OS) was 12.0 months (Figure 1b). Twelve patients discontinued treatment owing to an adverse event (interstitial pneumonia in 10 patients and peripheral neuropathy in two patients). The rate of patients who received second-line therapy was 58.3%.

### Prognostic variables

**Figure 2.**
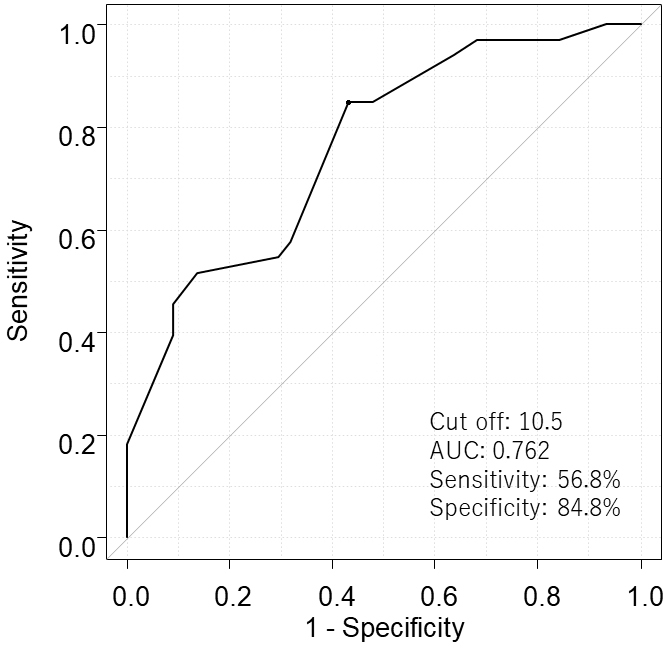
Receiver Operating Characteristic curve to set the optimal cutoff value for G8.

The median for G8 was 10.5. We performed ROC analysis and found a cutoff value of 10.5 [Area under the curve (AUC): 0.762; sensitivity: 56.8%; specificity: 84.8%] for the G8 was ideal for discriminating between patients’ survival (Figure 2). Thirty-four patients (44.2%) had low G8 (<10.5). The median for GNRI was 97.4, and 11 patients (14.3%) had low GNRI (<86). A total of 41 patients (54.5%) had high CEA (≥5.0), whereas 57 patients (74.0%) had high CA19-9 (≥37.0). The median baseline NLR was 2.66, and 12 patients (15.6%) had high NLR (≥3.9). Baseline elevated mGPS (1-2) was found in 18 patients (23.4%).

### Multivariate analysis to detect independent prognostic factors and ROC analysis

First, we explored prognostic factors (Table 3). ECOG PS 1 (p = 0.009), G8 <10.5 (p < 0.001), GNRI <86 (p = 0.05), initially reduced dose of GnP (p = 0.02), mGPS 1-2 (p = 0.04), and NLR ≥3.9 (p = 0.005) were extracted from the univariate analysis. A multivariate analysis was undertaken to identify pretreatment variables correlating with prognosis, revealing that ECOG PS 1 (HR 3.00, p = 0.02), NLR ≥3.9 (HR 2.72, p = 0.03), and G8 <10.5 (HR 5.38, p < 0.001) represented independent negative prognostic factors.

**Table 3.** Univariate and Multivariate Analyses to Detect Independent Prognostic Factors.

		Survival	Univariate	Multivariate	
Variable	n	month	*p* value	HR (95%CI)	*p* value
Gender					
Male	47	10.0	0.50		
Female	30	13.8			
ECOG performance status					
0	67	13.0	0.009	3.00(1.15-7.82)	***0.02***
1	10	8.1			
Disease status					
Locally advanced	21	13.5	0.12		
Distant metastasis	56	11.2	
G8 geriatric screening					
<10.5	30	8.0	<0.001	5.38 (2.76-10.5)	***<0.001***
≥10.5	47	18.5			
GNRI					
<86	11	8.1	0.05	0.63 (0.29-1.39)	0.26
≥86	66	13.0			
Initially reduced dose of GnP					
Yes	23	9.0	0.02	1.55 (0.86-2.80)	0.14
No	54	13.5			
NLR					
<3.9	65	13.0	0.005	2.72 (1.11-6.68)	***0.03***
≥3.9	12	7.6			
mGPS					
0	59	13.5	0.04	1.62 (0.81-3.26)	0.18
1-2	18	8.0			
CEA (ng/ml)					
<5.0	36	13.0	0.09		
≥5.0	41	11.0			
CA19-9 (U/ml)					
<37.0	20	14.6	0.79
≥37.0	57	11.5			

## Discussion

We revealed that ECOG PS, NLR, and G8 were independent prognostic factors in elderly patients with APC. Moreover, we identified the optimal cutoff value of the G8 score for predicting prognosis as 10.5.

G8 is simplified by extracting seven items from the Mini-Nutritional Assessment, one of the GA tools used to evaluate nutritional status. G8 comprises items to assess the functional status, nutrition, neuropsychological status, and polypharmacy. Consequently, G8 has a good prognostic value for OS, possibly because G8 confers the advantage of evaluating general patients’ conditions via multiple aspects. G8 score of ≤14 is considered abnormal ^(17)^, but 92% of patients had a G8 score of ≤14 in our and previous studies, demonstrating that most elderly patients with APC had an abnormal G8 score ^(20), (21)^. Particularly, pancreatic cancer is associated with an excessive catabolic state leading to cachexia, defined as significant weight loss depending on the prevalence of sarcopenia and individual BMI ^(22), (23)^. All patients in our study were in relatively general condition with ECOG PS of 0 or 1, tolerating GnP chemotherapy; thus, APC specific cutoff value for G8 is important. On the other hand, patients with a G8 score of less than 10.5 are considered to be in a pre-cachectic state, requiring supportive care, such as nutritional support, exercise therapy, and pharmacotherapy (e.g., ghrelin receptor agonist) along with GnP chemotherapy.

Studies have shown that higher NLR correlated with adverse survival outcomes in patients with APC ^(10), (11), (12), (13)^. Elevated neutrophil levels and relative lymphocytopenia often cause high NLR. High neutrophil levels can accelerate tumor cell progression by upregulating various inflammatory cytokines and providing a suitable microenvironment for tumor growth ^(24), (25)^. Furthermore, lymphocytopenia caused by many inhibitory immunological mediators secreted by tumor cells represents an immunosuppressive condition among cancer patients and contributes to a poorer outcome ^(26)^. Therefore, NLR reflects a balance between the tumor-promoting environment and the anti-tumor immune status.

**Figure 3.**
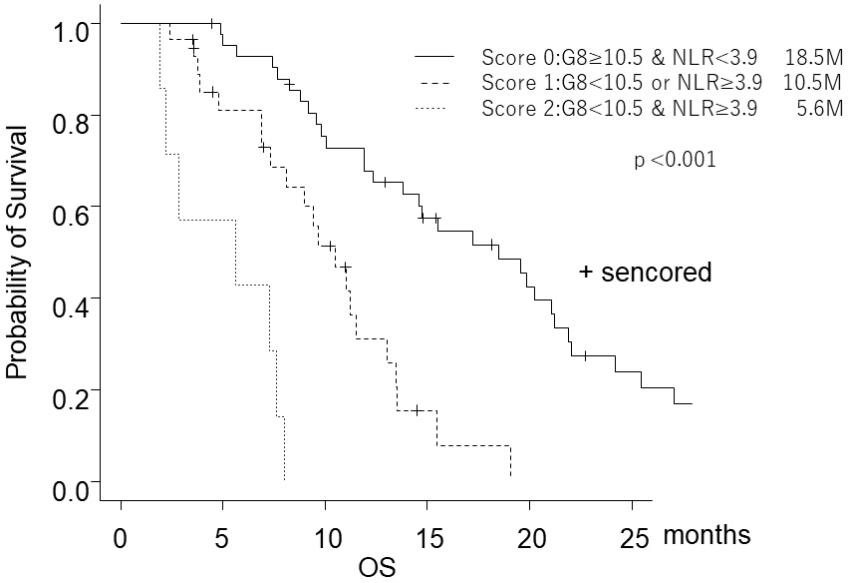
Kaplan-Meier estimates for overall survival according to three risk groups.

G8 is based on subjective evaluation, NLR is based on objective assessment, and ECOG PS and G8 are correlated ^(21)^. Thus, we thought combining G8 and NLR might be a better evaluation method. Our study showed that prognoses could be stratified by categorizing patients according to G8 and NLR (Figure 3). Elderly patients with high G8 and low NLR are “fit patients” who should be treated with the same chemotherapy standard as young patients. Elderly patients with low G8 and high NLR are “frail patients,” and the best supportive care may be appropriate. Elderly patients with either high G8 or low NLR might be “vulnerable patients,” less toxic treatment may be more appropriate than the standard treatment.

The limitations of this study included: (i) retrospective and single-center design; (ii) lacking consideration of the prognostic impact of second-line chemotherapy; and (iii) involvement of only the Japanese population.

In conclusion, we found that G8 represented an independent prognostic factor for survival with nab-paclitaxel plus gemcitabine in elderly patients with APC. Our results suggest that the evaluation of the factor is useful in prognosis prediction and chemotherapy adjustment. Thus, further validation in a prospective study is warranted.

## Article Information

### Conflicts of Interest

None

### Acknowledgement

The authors do not have commercial or other associations that might pose a conflict of interest, and no external funding was received for this study.

The authors thank Enago (www.enago.jp) for the English language review.

### Author Contributions

MK was the principal investigator, drafted the manuscript, and approved the final version of the manuscript. YM, YT, HY, TY, and KT contributed to data acquisition, reviewed the draft manuscript, and approved the final version. FA supervised the research project, reviewed the draft manuscript, and approved the final version of the manuscript.

### Approval by Institutional Review Board (IRB)

Approval code: R2-3

Name of institution: Kofu Municipal Hospital

Date of approval: 15 July 2020
